# Galangin, a natural flavonoid reduces mitochondrial oxidative damage in streptozotocin-induced diabetic rats

**DOI:** 10.1080/13510002.2017.1365224

**Published:** 2017-08-16

**Authors:** Amal A. Aloud, Chinnadurai Veeramani, Chandramohan Govindasamy, Mohammed A. Alsaif, Khalid S. Al-Numair

**Affiliations:** a Department of Food Sciences and Nutrition, College of Food and Agriculture Sciences, King Saud University, Riyadh, Saudi Arabia; b Department of Community Health Sciences, College of Applied Medical Sciences, King Saud University, Riyadh, Saudi Arabia

**Keywords:** Albino wistar rats, diabetes, mitochondria, lipid peroxidation, antioxidant

## Abstract

**Objective:** We designed this study to observe the effect of galangin on damaged mitochondria in the liver of diabetic rats.

**Methods:** Male albino Wistar rats were made diabetic by injecting streptozotocin (STZ) intraperitoneally (40 mg kg^−1^ body weight (BW)). Galangin (8 mg kg^−1^ BW) or glibenclamide (600 µg kg^−1^ BW) was given orally daily once for 45 days to both healthy and diabetic rats.

**Results:** Diabetic rats showed significant (*P* < 0.05) increase in liver mitochondrial oxidant [Thiobarbituric acid reactive substance (TBARS)] level and a significant decrease in enzymatic [superoxide dismutase (SOD), glutathione peroxidase (GPx)] and non-enzymatic (reduced glutathione (GSH)) antioxidant levels when compared with healthy rats. The mitochondrial enzymes isocitrate dehydrogenase (ICDH), alpha-ketoglutarate dehydrogenase (α-KGDH), succinate dehydrogenase (SDH) and malate dehydrogenase (MDH) and mitochondrial respiratory chain enzymes NADH-dehydrogenase and Cytochrome c-oxidase were decreased significantly (*P* < 0.05) in diabetic rats when compared with healthy rats. A natural flavonoid galangin administered to hyperglycemia-induced rats resulted in the following findings as compared to hyperglycemia-induced control rats: the oxidant levels decreased significantly (*P* < 0.05); the enzymatic and non-enzymatic antioxidant levels increased significantly (*P* < 0.05) and the function of mitochondrial enzymes and the mitochondrial respiratory chain enzymes increased significantly (*P* < 0.05).

**Conclusion:** From the results, we conclude that galangin could maintain liver mitochondrial function in diabetic rats.

## Introduction

Enhanced oxidative stress and decreased antioxidant status in diabetic patients and experimental animals contribute to the development of diabetic complications [1]. Prolonged hyperglycemia, increased production of free radicals, decreased antioxidant status, auto-oxidation of glycated proteins and increased lipid peroxidation play a major role in cellular apoptosis or necrosis in prolonged diabetic patients [2,3].

Streptozotocin (STZ)-induced damage to beta cells of pancreas results in diabetes in rats. The increased nitric oxide production, enhanced oxygen free radicals formation (OFRs) and enhanced protein glycation were caused by pancreatic β-cell damage in STZ-induced rats [4,5]. Also, STZ induction depleted antioxidant status in target cells, thereby enhancing the oxidative damage to the cells [6]. Prolonged hyperglycemia by STZ-induction caused apoptosis and necrosis in pancreatic cells and other target organs [5].

STZ-induced insulin-dependent diabetes mellitus can damage mitochondrial genome by increased free radical formation [7]. Thus, enhanced free radical production in mitochondria may damage β-cells, leading to chronic diabetic complications [8]. Eventual cellular death can occur by apoptosis or necrosis due to overwhelmed mitochondrial defense system and accumulation of the toxic compounds. STZ-elicited reactive oxygen species (ROS) leads to oxidative insult, which results in increased susceptibility of oxidative mitochondrial damage. Hence, it is apparent that STZ-induced damage might have diminished the proteins’ activities in mitochondria, which have changed the mitochondrial nature leading to mitochondrial damage in diabetic rats.

Recently, there have been resurgent interests in natural drug treatments to diabetes. Natural products from medicinal plants are growing public interest and pharmaceutical industry and researchers also have focused more for developing natural antidiabetic drug [9]. Natural products are safer because they are more harmonious with biological systems [10,11]. Flavonoids are natural polyphenolic compounds found in various plants in appreciable quantities. Several flavonoids from plants possess antioxidant, antidiabetic, anti-inﬂammatory, vasodilatory and anti-carcinogenic properties and others [12–15].

**Figure 1.**
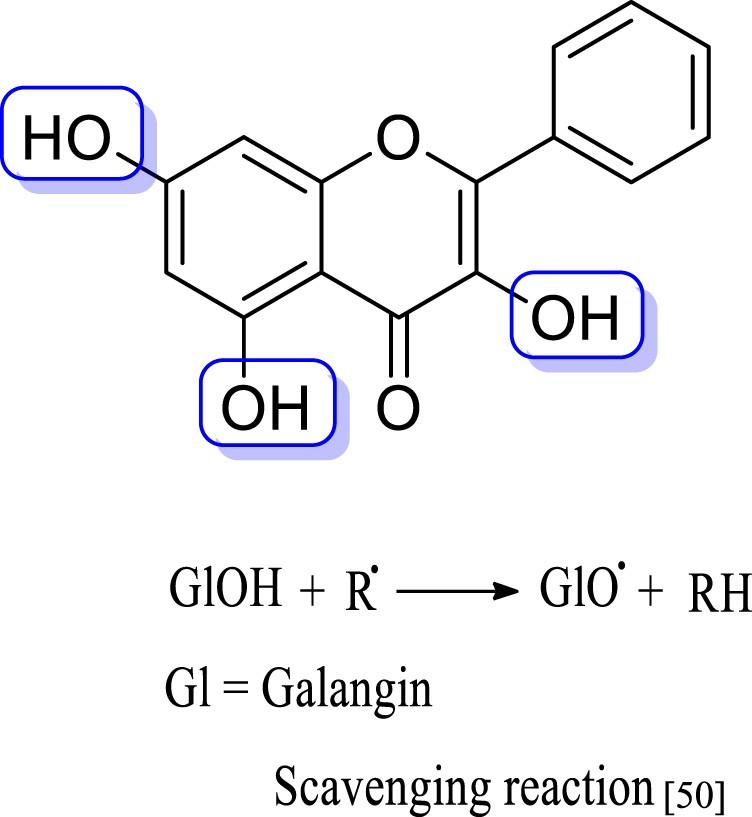
Galangin structure.

A natural flavonoid of galangin (3, 5, 7-trihydroxyﬂavone; Figure 1) is present in *Alpinia ofﬁcinarum Hance* root and honey [16]. Galangin has anti-inflammatory [17], antiviral [18], anti-clastogenic [19] properties, etc. and it also ameliorates certain types of cancers [20–23]. A previous study reported that the galangin has therapeutic effect against ischemic injury through the inhibition of mitochondrial dysfunction and mitochondrial cell death pathway [24]. Sivakumar and Anuradha revealed that galangin prevents oxidative and inflammatory changes in fructose-fed rats [25]. Recently, we reported that the galangin reduces oxidative stress and enhances antioxidant in STZ-induced hyperglycemia [26].

Oxidative stress is implicated in persistent complications of diabetes and galangin reduces hyperglycemia. We have designed this study to observe the galangin effect on oxidative mitochondrial damage in normal and STZ-induced hyperglycemic rats. Glibenclamide can motivate the insulin production from β-cells of pancreas (insulin secretagogue) without any detrimental effect. Hence, glibenclamide is commonly used by researchers for standard antidiabetic agent in animal model. Therefore, in our study also, galangin was matched with glibenclamide in terms of its effectiveness as a standard antidiabetic agent.

## Materials and methods

### Experimental animals

The healthy male Wistar rats weighting between 180and 200 g were obtained from the King Saud University Central Animal House. The obtained animals were housed in an air-conditioned room at 25 ± 1°C and exposed to day–night cycle during the experiments. The animals were provided normal laboratory pellet diet by *ad libitum*. The animal care policy was in accordance with the King Saud University Research Centre.

### Drug and chemicals

Galangin and STZ were obtained from Sigma–Aldrich (USA). Other analytical grade chemicals were obtained from various commercial suppliers.

### Experimental induction of diabetes

Hyperglycemia was induced into 12 h fasted healthy animals by an intraperitoneal insertion of single low dose of STZ (40 mg kg^−1^ BW) prepared with a fresh citrate buffer (0.1 M, pH 4.5). Once STZ was injected, 20% glucose was given for 24 h with drinking water for preventing mortality. Animals’ diabetes was confirmed by checking the plasma glucose after 96 h of injecting STZ. More than 220 or 220 mg dl^−1^ of plasma glucose was chosen for this experimental study.

### Experimental preparation

Six rats per group were included in five groups. Galangin (8 mg kg^−1^ dissolved with 5% DMSO) or 600 µg kg^−1^ glibenclamide (dissolved with DMSO (5%)) was given post orally daily once for 45 days. Recently, we reported that the dose determination study of galangin has three different quantities (4, 8 and 16 mg kg^−1^) in STZ-induced diabetic rats [26]. Eight mg kg^−1^ BW showed maximum improvement of glucose when compared with other doses. Hence, 8 mg kg^−1^ BW of galangin was finalized as an active dose and was used in the current study.

**Table:** 

Group 1:	Healthy normal rats were treated only 5% DMSO
Group 2:	Healthy normal rats were treated 8 mg kg^−1^ of galangin
Group 3:	STZ-induced hyperglycemic control rats
Group 4:	Hyperglycemic rats were treated 8 mg kg^−1^ of galangin
Group 5:	Hyperglycemic rats were treated 600 µg kg^−1^ of glibenclamide

After finishing the treatment period, the 12 ho fasted animals were killed by cervical dislocation for lack of sensation or feeling, which was made by intramuscular injection of ketamine 24 mg kg^−1^ BW. Liver tissue was collected and used for isolation of mitochondria.

### Biochemical assays

By the standard method of Johnson and Lardy, the mitochondria (liver tissue) were isolated [27]. The thiobarbituric acid reactive substance (TBARS) concentration in liver mitochondria was analyzed by the method of Niehaus and Samuelsson[28]. The activity of superoxide dismutase (SOD) and GPx were assessed by the methods of Kakkar et al. [29] and Rotruck et al. [30] respectively in liver mitochondria. The level of GSH from liver mitochondria was estimated by the method of Ellman [31]. The activities of ICDH, α-KGDH, SDH and MDH were assayed according to the method of Bell and Baron [32], Reed and Mukherjee [33], Slater and Bonner [34] and Mehler et al. [35] respectively. Cytochrome-c-oxidase enzyme was assessed by the method of Pearl et al. [36]. NADH-dehydrogenase enzyme was determined by the method of Minakami et al. [37].

### Statistical study

The statistical study was carried out with SPSS software package (9.05). The results were examined by DMRT and ANOVA. The results were shown in average of six rats per group as mean ± S.D. The significant was considered in *P* values <0.05.

## Results

### Effects of galangin on liver mitochondrial TBARS, GSH, SOD and GPx

**Figure 2.**
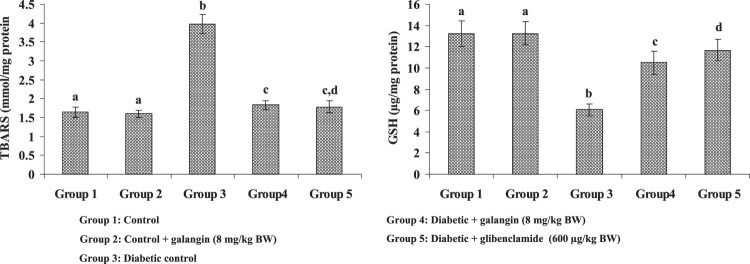
Effect of galangin on liver mitochondrial TBARS and GSH concentrations of STZ-caused hyperglycemic rats. Data are means ± SEM, *n* = 6. Groups 1 and 2 significantly are not different (a, a) (*P* < 0.05). Groups 4 and 5 are different significantly compared to group 3 (b vs. c, cd, d) (*P* < 0.05). U^a^ – Enzyme concentration required for 50% inhibition of NBT reduction/min s. U^b^ – µmol of reduced glutathione consumed/min.

**Figure 3.**
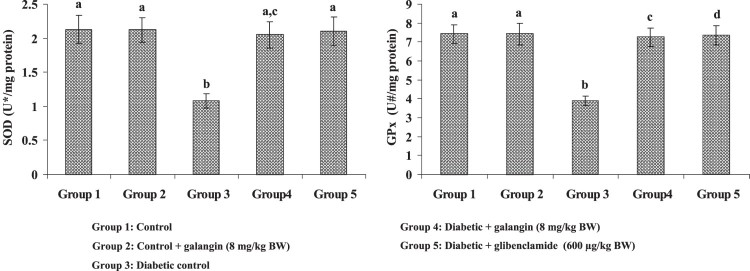
Effect of galangin on liver mitochondrial enzymic antioxidants of STZ-caused hyperglycemic rats. Data are means ± SEM, *n* = 6. Groups 1 and 2 significantly are not different (a, a) (*P* < 0.05). Groups 4 and 5 are different significantly compared to group 3 (b vs. ac, a, c, d) (*P* < 0.05). U* – Enzyme concentration required for 50% inhibition of NBT reduction/min s. U^#^ – µmol of reduced glutathione consumed/min.

Figures 2 and 3 show the TBARS concentration and enzymic (SOD, GPx) and other (GSH) antioxidants in hepatic mitochondria of healthy and STZ-induced hyperglycemic rats. A liver mitochondrial TBARS concentration had elevated more significantly in diabetic rats than in healthy rats. Galangin drug decreased the mitochondrial TBARS level significantly in hyperglycemic rats when compared with healthy rats. The enzymic (SOD, GPx) and other (GSH) antioxidants decreased significantly in hyperglycemic rats when compared with healthy rats. Galangin drug administration to hyperglycemic rats, the enzymic SOD and GPx in liver mitochondria significantly increased and other antioxidant GSH significantly increased in mitochondria of liver to near control rats.

### Effects of galangin on liver mitochondrial tricarboxylic acid cycle (TCA) enzymes’ activities

**Figure 4.**
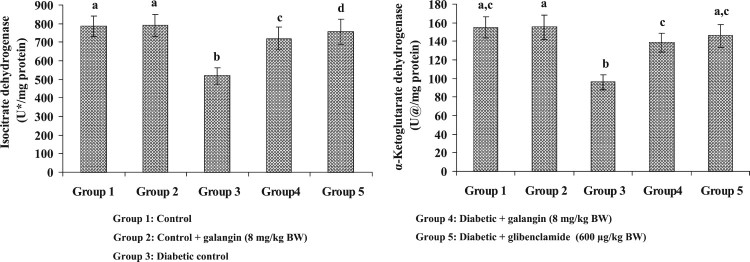
Effect of galangin on liver mitochondrial isocitrate dehydrogenase (ICDH) and alpha-ketoglutarate dehydrogenase (*α*-KGDH) of STZ-caused hyperglycemic rats. Data are means ± SEM, *n* = 6. Groups 1 and 2 significantly are not different (a, a) (*P* < 0.05). Groups 4 and 5 are different significantly compared to group 3 (b vs. c, d, ac) (*P *< 0.05). U* – nmol of α-ketoglutarate formed/h. U@ – nmol of ferrocyanide formed/h.

**Figure 5.**
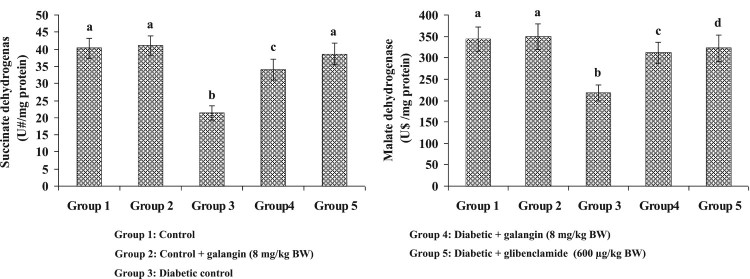
Effect of galangin on liver mitochondrial succinate dehydrogenase (SDH) and malate dehydrogenase (MDH) of STZ-caused hyperglycemic rats. Data are means ± SEM, *n* = 6. Groups 1 and 2 significantly are not different (a, a) (*P *< 0.05). Groups 4 and 5 are different significantly compared to group 3 (b vs. c, a, d) (*P* < 0.05). U# – nmol of succinate oxidized/min. U$ – nmol of NADH oxidized/min.

Figures 4 and 5 show the mitochondrial TCA enzymes’ activities in liver mitochondria of healthy and hyperglycemic rats. The enzymes’ activities in mitochondria (α-KGDH, ICDH, MDH and SDH) were decreased considerably in hyperglycemic rats when compared with healthy rats. Galangin drug had increased these TCA cycle enzymes’ activities to near-healthy normal rats.

### Effects of galangin on liver mitochondrial respiratory chain enzymes’ activities

**Figure 6.**
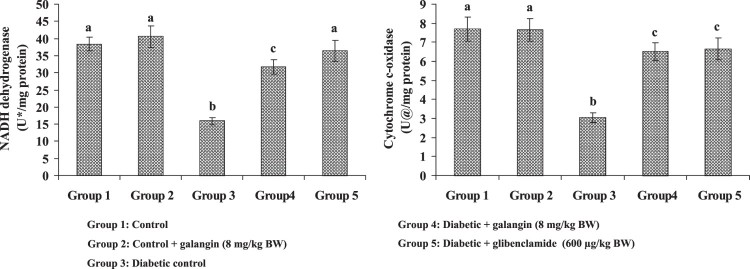
Effect of galangin on liver mitochondrial respiratory chain enzymes of STZ-caused hyperglycemic rats. Data are means ± SEM, *n* = 6. Groups 1 and 2 significantly are not different (a, a) (*P* < 0.05). Groups 4 and 5 are different significantly compared to group 3 (b vs. c, a) (*P* < 0.05). U* – nmol of NADH oxidized/min. U^@^ – change in OD x 10^−2^/min.

Figure 6 shows the activity of respiratory chain enzymes’ in mitochondria (liver) of healthy and hyperglycemic rats. The NADH-dehydrogenase and Cytochrome c-oxidase decreased enzymes’ activities in hyperglycemic rats when compared with healthy rats. Galangin drug had improved the respiratory chain enzymes’ activities in hyperglycemic rats to the level seen in healthy rats.

## Discussion

Mitochondria, which play a crucial responsibility in cell functions, appear to constitute one of the main sources of free radicals [38,39]. Nearly 2% of the total mitochondrial oxygen consumption results in ROS generation [40,41]. The enhanced ROS generation is extensively affected in mitochondrial functions. The inner mitochondrial membrane and even physiological conditions also are affected by oxidative stress due to the high content of polyunsaturated fatty acids (PUFA) [38,40]. The ROS produced during electron transport chain reacts with PUFA, which leads to peroxidation of membrane lipids and results in altering the mitochondrial membranes’ structure, irretrievable swelling, disruption and abnormal mitochondrial functions [41–43]. Oxidative stress is an important source to the pathogenesis of many diseases including diabetes mellitus. In this study, the TBARS concentrations in liver mitochondria increased remarkably in hyperglycemic rats when compared with healthy rats. Thus, increased level of TBARS in liver mitochondria indicates lipid peroxidation, which may alter the structural integrity of membranes in mitochondria resulting in mitochondrial dysfunction. Administration of galangin to hyperglycemic rats decreased the TBARS concentrations to those of normal rats. In an earlier study, galangin decreased oxidative status and increased antioxidant status [26]. Thus, the antioxidative action of galangin may have an excellent property to scavenge the free radicals.

Antioxidants play a vital role in protecting the human body from the oxidative stress by the scavenging mechanism of free radicals [44]. Mitochondria may limit the ROS effects by antioxidants, including SOD, CAT, GPx, GSH, vitamin E and vitamin C [45]. In this study, the enzymic (SOD and GPx) and non-enzymic (GSH) antioxidants decreased mitochondria of hyperglycemic rats remarkably when compared with normal healthy rats. Thus, decreased antioxidant status in liver mitochondria might be due to increased utilization by tissues or decreased synthesis. Galangin increased the antioxidant level in hyperglycemic rats when compared with that in normal healthy rats. Thus, observed results indicate that the galangin improves antioxidant status in liver mitochondria, which potentially reduces the membrane lipid peroxides. Some antioxidants have potential effects to the treatment of oxidative stress-related diseases including diabetes mellitus [46,47]. A recent study suggests that increased intake of antioxidants can avoid or minimize diabetic complications [48]. Flavonoids possess various beneficial biological effects that include antioxidant activity [49]. The dietary flavonoids can scavenge the radical by providing hydrogen from their hydroxyl group [50]. Previous reports have also revealed that hydroxyl groups play a vital role in antiradical activity [50,51]. Galangin (3,5,7-trihydroxyflavone) can provide easily hydrogen form their 3,5,7-trihydroxyl group and scavenge free radicals [50]. The present study reveals that galangin enhances the antioxidant property in hyperglycemic rats. This might be due to their scavenging property. Sivakumar and Anuradha [25] reported that galangin protects cellular antioxidants in fructose-fed rats.

Mitochondrial damage has occurred during diabetic complications in liver [52]. STZ-elicited ROS leads to oxidative insult, which may be the reason for the oxidative damage in mitochondrial proteins. Therefore, prolonged hyperglycemia has enhanced the ROS generation and lipid peroxides, which would diminish the mitochondrial proteins as well as mitochondrial function and ultimately cause toxic effects in the mitochondrion of liver, heart and kidney. We observed that decreased liver mitochondrial enzymes α-KGDH, SDH, MDH and ICDH in hyperglycemic rats. Thus, decreased TCA cycle enzymes’ activities might have attributed to the increased ROS production in hyperglycemic rats. The previous study demonstrated that the ROS is involved in mitochondrial damage [53]. Galangin treated to diabetic rats, the activity of TCA cycle enzymes significantly reversed to near-normal healthy rats. Thus, results indicate that galangin may improve the antioxidant property in mitochondria, and minimize the mitochondrial damage in the liver.

NADH-dehydrogenase constitutes complex I of the electron transport chain, which passes electron from NADH to coenzyme Q. Cytochrome c-oxidase provides electrons directly to molecular oxygen and constitutes complex IV. The activities of these enzymes have been decreased in the diabetic state due to the increased formation of ROS and cellular damage [54]. These decreased activities of enzymes in hyperglycemic rats could be due to the inhibition of electron flow from NADH to oxygen. Administration of galangin significantly increased the activities of these enzymes in hypertensive rats due to its free radical scavenging effect.

In conclusion, galangin could maintain liver mitochondrial function in hyperglycemic rats. The possible mechanism for the observed positive results of galangin could be due to improved antioxidant status. Furthermore, the possible mechanism of galangin is needed to establish in future.
